# Unveiling the cognitive fog in lung cancer patients: non-invasive exploration of blood–brain barrier disruption and brain structural changes

**DOI:** 10.1080/07853890.2026.2662776

**Published:** 2026-06-18

**Authors:** Da-Fu Zhang, Huan Ma, Zhi-Ping Zhang, Jing Ai, Wen-Ting Cao, Zhen-Hui Li

**Affiliations:** a Department of Radiology, The Third Affiliated Hospital of Kunming Medical University, Yunnan Cancer Hospital, Yunnan Cancer Center, Kunming, Yunnan, China; b Department of Dermatology, The Second Affiliated Hospital of Kunming Medical University, Kunming, Yunnan, China

**Keywords:** Blood–brain barrier, brain structure, cognitive, dynamic contrast-enhanced MRI, lung cancer

## Abstract

**Background:**

This study investigates the correlation between blood–brain barrier (BBB) damage and cognitive impairment in patients suffering from untreated lung cancer. The study aims to understand the impact of BBB permeability on cognitive function and brain structure in this patient population.

**Patients and methods:**

This study included 104 lung cancer patients and 40 healthy individuals as controls. The permeability of the blood–brain barrier was evaluated using dynamic contrast-enhanced magnetic resonance imaging (DCE-MRI). The study analyzed the correlation between BBB permeability and both brain structure and cognitive function to determine the extent of BBB damage’s influence on cognitive impairment.

**Results:**

The study revealed a significant increase in BBB permeability among lung cancer patients, particularly in those experiencing cognitive impairment. Those with cognitive impairment exhibited a substantial decrease in cortical volume across several brain regions. Notably, the *K*^trans^ value in the right superior parietal cortex was significantly elevated in the cognitive impairment group compared to both the non-cognitive impairment group and the healthy control group.

**Conclusion(s):**

The findings indicate a strong association between BBB damage and cognitive impairment in lung cancer patients. DCE-MRI has shown promise as an effective diagnostic tool for assessing BBB damage in the context of lung cancer treatment. This research contributes novel insights into the pathophysiological mechanisms underlying cognitive impairment in lung cancer patients and provides valuable information for the development of personalized therapeutic strategies.

## 1. Introduction

In recent years, lung cancer has become the leading cause of cancer morbidity and mortality worldwide [1]. chemotherapy remains a primary treatment strategy for prolonging patient survival, but it may lead to chemotherapy-related cognitive impairment (CRCI) [2]. CRCI primarily affects learning, memory, and task performance, often referred to as ‘chemo brain,’ which significantly impacts patients’ quality of life. Current neuroimaging studies have focused on exploring treatment-related cognitive changes, while few have investigated cognitive impairment in pre-treatment patients [3,4]. Studies show that 20% to 30% of patients exhibit cognitive impairment before receiving any systemic therapy, suggesting that cancer itself may induce early cognitive impairment, but specific risk factors remain unknown [5]. Cognitive function is more likely to decline in cancer patients than in non-cancer populations [6]. Research indicates that the incidence of dementia is significantly higher in cancer patients than in non-cancer populations, highlighting the severity of the issue [7].

Neuroimaging studies have revealed the effects of chemotherapy on brain structure, mainly in the form of early gray matter reduction and white matter degeneration [8,9]. A study by Joly et al. noted that post-chemotherapy decline in executive and memory functions in cancer patients was associated with a reduction in gray matter in frontal regions [10]. In patients with non-small cell lung cancer, cognitive deficits after chemotherapy are particularly significant, mainly manifesting as structural differences in gray and white matter in bilateral regions of the limbic system [11].

**Figure 1.**
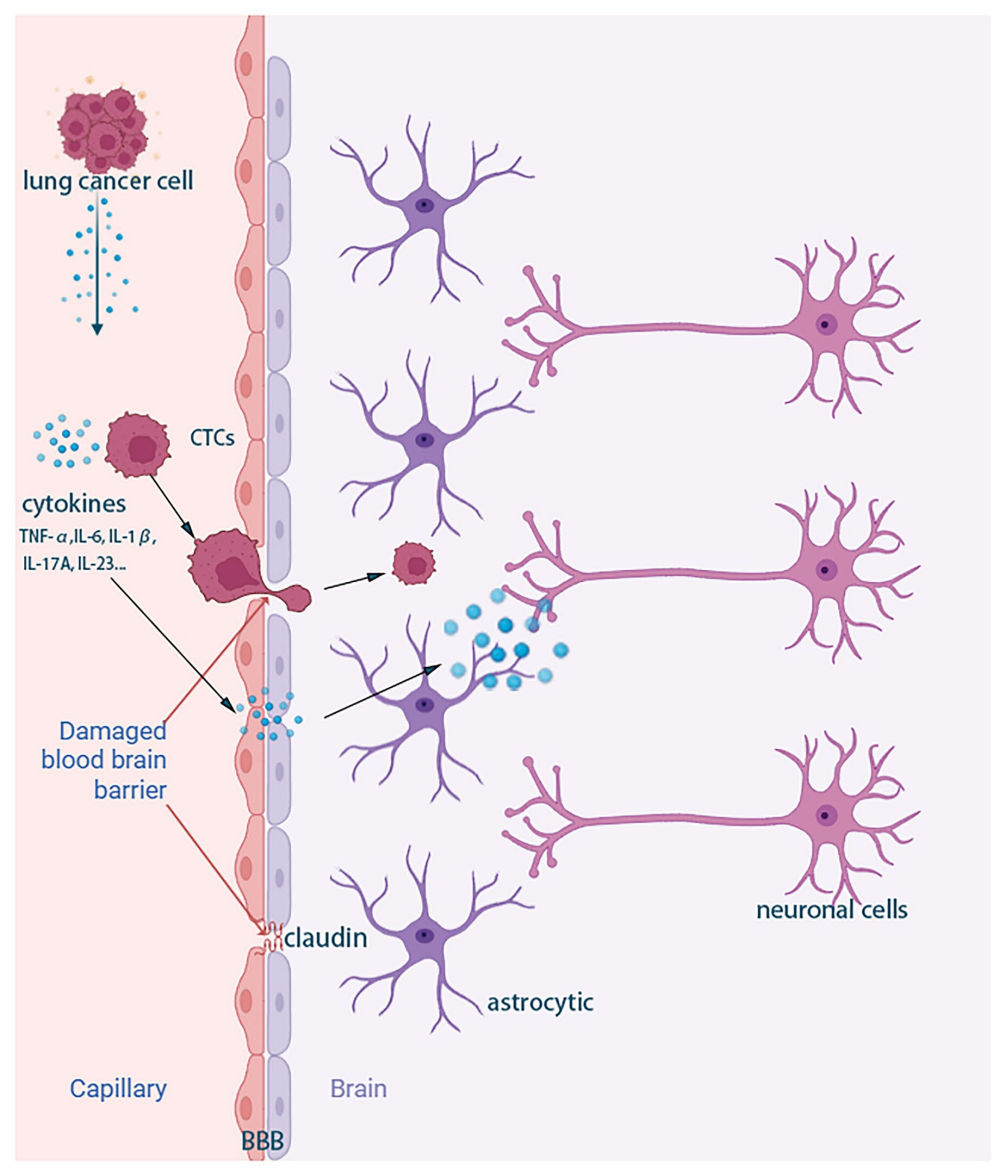
Proposed mechanism of lung cancer-induced blood–brain barrier disruption and neuroinflammation.

Studies of the underlying pathophysiological mechanisms of CRCI have identified several key mechanisms. One is the role of pro-inflammatory cytokine [12], which are directly triggered by the tumor and its progression, such as the release of tumor necrosis factor alpha (TNF-α), interleukin 6 (IL-6), IL-1β, IL-17A, and IL-23 [13,14]. These pro-inflammatory factors can signal the brain through multiple pathways, triggering changes in neurotransmitter function and brain circuitry [15]. Another mechanism involves the invasion of lung cancer tumor cells into the circulatory system to form circulating tumor cells (CTCs), which are mesenchymal-transforming cells capable of secreting cytokines that lead to the downregulation of proteins associated with the maintenance of the integrity of the blood–brain barrier (BBB) and increase the permeability of the BBB [16]. The BBB normally restricts the entry of most blood-borne inflammatory and tumor factors into the brain to maintain normal neuronal activity. Disruption of the BBB allows penetration of these factors into the brain, causing a neuroinflammatory response that triggers multiple neurological dysfunctions and degenerative pathways [17]. In other disorders, such as traumatic brain injury, epilepsy, Alzheimer’s disease, bipolar disorders, and systemic lupus erythematosus, impairment of the blood–brain barrier function has been shown to lead to neuronal damage [18–20]. These pathophysiological mechanisms may interact to affect brain structure and function, resulting in cognitive impairment (Figure 1).

Lung cancer cells and circulating tumor cells (CTCs) release pro-inflammatory cytokines (e.g. TNF-α, IL-6, IL-1β, IL-17A, IL-23). These cytokines target the capillary endothelium constituting the blood–brain barrier (BBB), leading to its disruption. A compromised BBB allows cytokines and potentially other mediators to infiltrate the brain parenchyma, where they can activate astrocytes and directly or indirectly harm neuronal cells, potentially contributing to neuroinflammation and associated neurological complications.

BBB dysfunction or disruption plays a key role in the pathogenesis of various brain disorders; therefore, accurate monitoring of BBB changes is crucial for the diagnosis and treatment of disease. Dynamic contrast-enhanced magnetic resonance imaging (DCE-MRI) provides a noninvasive means of measuring the level of microleakage of the BBB [21]. Cramer and Larsson’s study [22] pointed out that at low leakage rates, i.e. leakage rates below 0.3 ml/100g/min (approximately equal to 0.003 min^−1^), the Patlak model is recommended for analysis. BBB permeability is quantified by the parameter *K*^trans^, a widely used method for assessing BBB function. DCE-MRI has proven its value in monitoring BBB disruption in multiple sclerosis, bipolar disorder, systemic lupus erythematosus, and brain tumors [19,20,23,24].

Based on the hypothesis that cognitive impairment may be associated with BBB impairment in untreated lung cancer patients, this study aims to explore the potential relationship between cognitive impairment and BBB impairment in lung cancer patients.

## 2. Materials and methods

This study employs a prospective design. From June 2021 to December 2022, consecutive cases fulfilling the inclusion criteria were collected at the Third Affiliated Hospital of Kunming Medical University. The study was formally approved by the Ethics Committee of the Third Affiliated Hospital of Kunming Medical University (NO. SLKYLX202118), ensuring ethical and legal research conduct. All participating patients signed informed consent forms and fully understand the objectives, processes, potential risks, and benefits, thereby safeguarding their rights and interests.

This study was a cross-sectional analysis of untreated lung cancer patients (LCs) to evaluate their brain structure *via* brain scans and compare them with healthy controls (HCs) matched for age, sex, and smoking habits. The study period was from June 2021 to December 2022, and was conducted at the Department of Thoracic Surgery, Third Affiliated Hospital of Kunming Medical University. During this period, 104 untreated lung cancer patients and 40 healthy controls were enrolled. Healthy controls were matched to lung cancer patients by age, sex, and smoking history. All lung cancer patients were included before pathological diagnosis and without any treatment (including surgery, chemotherapy, radiotherapy, or immunotherapy). According to the TNM staging system (eighth edition) [25], lung cancer patients were categorized into early stage (stage I) and advanced stage (stages II to IV). Healthy controls were recruited *via* online advertisements and were matched to patients by age, sex, and smoking history. Exclusion criteria for HCs included a history of any cancer, neurological or psychiatric disorders, chronic systemic diseases (e.g. hypertension, diabetes), significant visual or hearing impairment, and use of psychotropic medications. All HCs were from the same geographical region (Kunming, Yunnan Province) to minimize environmental and genetic confounding factors.

**Figure 2.**
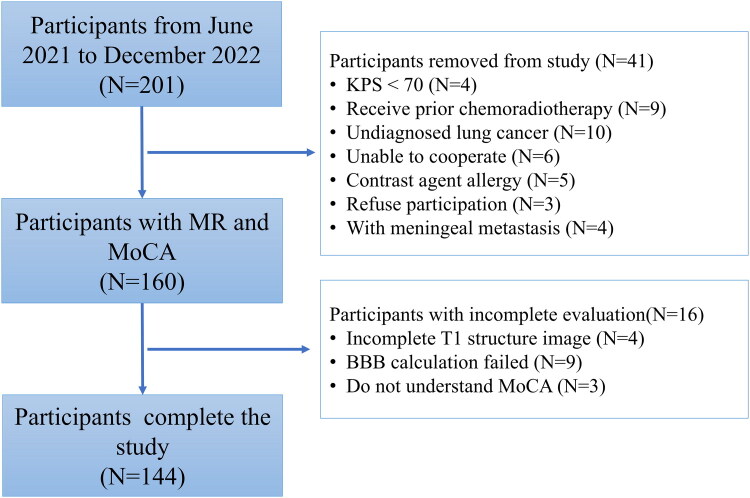
A workflow diagram for cases selection. KPS, Karnofsky performance status; MoCA, Montreal cognitive assessment; BBB, blood–brain barrier.

Some participants were excluded from the analysis due to difficulties with scanning, allergies to contrast agents, or data calculation errors. Exclusion criteria included: prophylactic brain irradiation, presence of brain metastases, a known history of stroke, brain trauma, epilepsy, hypertension, diabetes, Alzheimer’s disease, Parkinson’s disease, other acute mental or neurological disorders, a history of significant medical conditions (e.g. anemia, severe heart disease, thyroid dysfunction, or liver and kidney dysfunction), and severe vision or hearing loss (Figure 2).

### 2.1. MRI acquisition

All MRI data were collected using a 3.0 T Discovery MR750 scanner (GE Healthcare, Waukesha, Wisconsin, USA). To enhance image quality, we utilized a 21-channel head and neck coil. To minimize head movement during scanning, we employed a snug yet comfortable foam pad and provided earplugs and headphones to mitigate scanner noise. Each participant underwent the following sequence of scans: Conventional MRI sequences, including T2-weighted imaging, T1-weighted imaging, T2FLAIR imaging, and T1-weighted fat suppression enhanced imaging, were conducted to exclude significant brain lesions or brain metastases (Refer to Supplementary Table 1 for details).

#### 2.1.1. 3D high-resolution T1 imaging

A high-resolution 3D T1-weighted axial scan technique was employed with the following parameters: Bravo scan technique, echo time (TE) of 3.3 ms, repetition time (TR) of 8.6 ms, field of view (FOV) of 256 × 256 mm, acquisition matrix of 256 × 256, voxel size of 1 × 1 × 1 mm³, bandwidth of 31.25 kHz, flip angle (FA) of 12°, acceleration factor of 2, an average of 2 excitations, and a total scan time of approximately 270 s.

#### 2.1.2. DCE imaging

The 3D-SPGR sequence (3-D spoiled gradient echo sequence) was used, with flip angles set at 5° and 14°, respectively, and 5 scanning periods at each angle, each lasting about 13 s. At the start of the fifth period with a flip angle of 14°, a contrast enhancement agent was administered *via* high-pressure injection into the antecubital vein (gadopentetate meglumine, dose 0.05 mmol/kg) at a flow rate of 3.0 ml/s. Immediately post-injection, an equal volume of saline was injected at the same rate to maintain dynamic contrast scanning (Supplementary Table 1).

### 2.2. Cortical segmentation

Cortical segmentation was performed using FreeSurfer 7.2 (https://surfer.nmr.mgh.harvard.edu/)., a widely-used software for brain image analysis. This tool automatically identifies and measures brain regions based on the Desikan-Killiany atlas, a standardized map of brain structures, allowing us to calculate cortical volumes in 35 key regions linked to cognitive functions. All cortical volumes were normalized to total intracranial volume (TIV) to account for inter-individual differences in brain size, including age-related atrophy.

### 2.3. BBB data preprocessing and calculation

Previous studies have shown that the Patlak model offers superior accuracy for assessing diseases associated with BBB microdamage. Specifically, when quantifying BBB microleakage using dynamic contrast-enhanced magnetic resonance imaging (DCE-MRI), the Patlak model has consistently demonstrated excellent reproducibility. Leveraging these findings, this study employed the Patlak model to determine the volume transfer constant *K*^trans^ (min^−1^), serving as a metric for BBB leakage. To calculate *K*^trans^, we utilized SPM12 software (University College London, SPM12<http://www.fil.ion.ucl.ac.uk/spm&gt) to align and normalize the serial T1-weighted images post-contrast injection to the Montreal Neurological Institute (MNI) template. The images were co-registered to an average image, employing a 14-degree flip angle to account for head movement. Subsequently, we applied the Patlak graphical analysis, integrating T1 signal intensity maps with the arterial input function (AIF) to calculate *K*^trans^. The AIF was derived by identifying the region adjacent to the superior sagittal sinus as the region of interest (ROI). The calculation of *K*^trans^ was performed in a MATLAB environment. Finally, the brain was segmented into 116 distinct regions based on the Anatomical Automatic Labeling (AAL) atlas, and the mean *K*^trans^ values for each region, as well as for the entire brain in gray and white matter, were statistically analyzed.

### 2.4. Cognitive function evaluation

Currently, no universally accepted method exists for screening CRCI that is both valid and reliable. However, the Montreal cognitive assessment (MoCA) scale has been proposed as a more suitable tool for evaluating cognitive function in oncology patients. In this study, we selected the Beijing version of the MoCA scale to assess the cognitive function of participants. This version includes cultural and linguistic adaptations for the Chinese population, such as the substitution of certain verbal memory items and visuospatial tasks to better align with local cognitive norms. These modifications enhance its sensitivity and specificity for detecting mild cognitive impairment in Chinese oncology patients, thereby improving the validity of cognitive assessment in this cohort.

### 2.5. Statistical analysis

In this study, we utilized various statistical methods for data analysis and interpretation. For continuous variables, we employed ANOVA or Kruskal–Wallis tests to determine if there were significant differences in group means. These tests are ideal for comparing means across three or more independent samples. For categorical data, we applied the chi-square (χ^2^) test, a standard approach for evaluating statistical relationships between two or more categorical variables. In comparisons of cortical volumes and *K*^trans^ values, age was included as a covariate in sensitivity analyses to control for its potential confounding effect.

All statistical analyses were conducted using R, a prevalent programming language and software environment renowned for its comprehensive suite of statistical methods and graphical tools, facilitating efficient data processing and analysis.

Graphical representations were generated using R integrated with GraphPad Prism software (version 9.0.0, GraphPad Software, Inc., San Diego, California). GraphPad Prism is a popular tool for scientific research and clinical trials, offering a user-friendly interface and a variety of charting options to aid in the clear presentation and interpretation of data.

For multiple comparisons, Bonferroni correction was applied separately to two families of tests: (1) cortical volume comparisons across 35 Desikan–Killiany regions, and (2) *K*trans comparisons across 116 AAL atlas regions. Statistical significance was declared at adjusted thresholds of α = 0.05/35 ≈ 0.00143 for cortical volume analyses and α = 0.05/116 ≈ 0.00043 for *K*trans analyses. This approach minimizes the likelihood of spurious findings due to multiple comparisons, thereby bolstering the robustness and reliability of our study’s outcomes.

## 3. Results

### 3.1. Demographic data

A total of 104 patients with lung cancer and 40 healthy controls were enrolled in this study. No significant differences were observed between the groups in terms of age, sex, Karnofsky Performance Status (KPS) score, years of education, and smoking history (Table 1).

**Table 1.** Demographics of lung cancer and HC groups.

	HCs (*n* = 40)	LCs (*n* = 104)	*χ^2/^t*	*P*值
Gender				
Male/Female	20/20	55/49	0.096	0.853^a^
age (* $\bar{x}$ ± s*), year	52.28 ± 7.44	55.02 ± 8.06	−1.869	0.064^b^
smoking (%)	15/40	33/104	0.433	0.556^a^
KPS score (* $\bar{x}$ ± s*)	95.25 ± 7.51	93.65 ± 11.66	0.803	0.423^b^
education (* $\bar{x}$ ± s*), year	9.22 ± 2.88	8.24 ± 4.52	1.286	0.200^b^

Data are presented as mean ± standard deviation (*x ± s*), number (*n*) with percentage (%), or interquartile range (IQR). Statistical significance was assessed using the chi-square (χ²) test for categorical variables and the two-sample *t* test for continuous variables. No statistically significant differences were observed among the indicators in the table.

KPS, Karnofsky Performance Status.

### 3.2. Clinical data and cognitive scale evaluation of LCs

Of the 104 LC patients, 51.9% were diagnosed with early-stage lung cancer (stage I), 68.3% had adenocarcinoma, and 21.2% had tumors with a diameter greater than 5 cm. Regarding cognitive function, 32.7% of the LC patients exhibited mild cognitive impairment. None of the patients had received prior treatment, and there were no instances of brain metastasis (Table 2).

**Table 2.** Clinical data of LCs.

	*N*(*n* = 104)	%
Clinical stage		
I	52	50
II	6	5.8
III	25	24
IV	21	20.2
Pathological pattern		
Squamous carcinoma	24	23.1
Adenocarcinoma	68	65.4
Small cell adenocarcinoma	12	11.5
Serum markers		
CEA ≥ 3.4	45	43.3
NSE ≥ 16.3	24	23.1
CYFRA21-1 ≥ 16	38	36.5
SCC ≥ 1.5	16	15.4
Maximum tumor diameter		
≥5cm	22	21.2
Cognitive scores		
MoCA < 25	34	32.7

CEA, carcinoembryonic antigen; NSE, neuron-specific enolase; CYFRA21-1, cytokeratin 19 fragment; SCC, squamous cell carcinoma antigen; MoCA, Montreal cognitive assessment.

### 3.3. Changes of cerebral cortex volume in lung cancer patients with cognitive impairment

**Figure 3.**
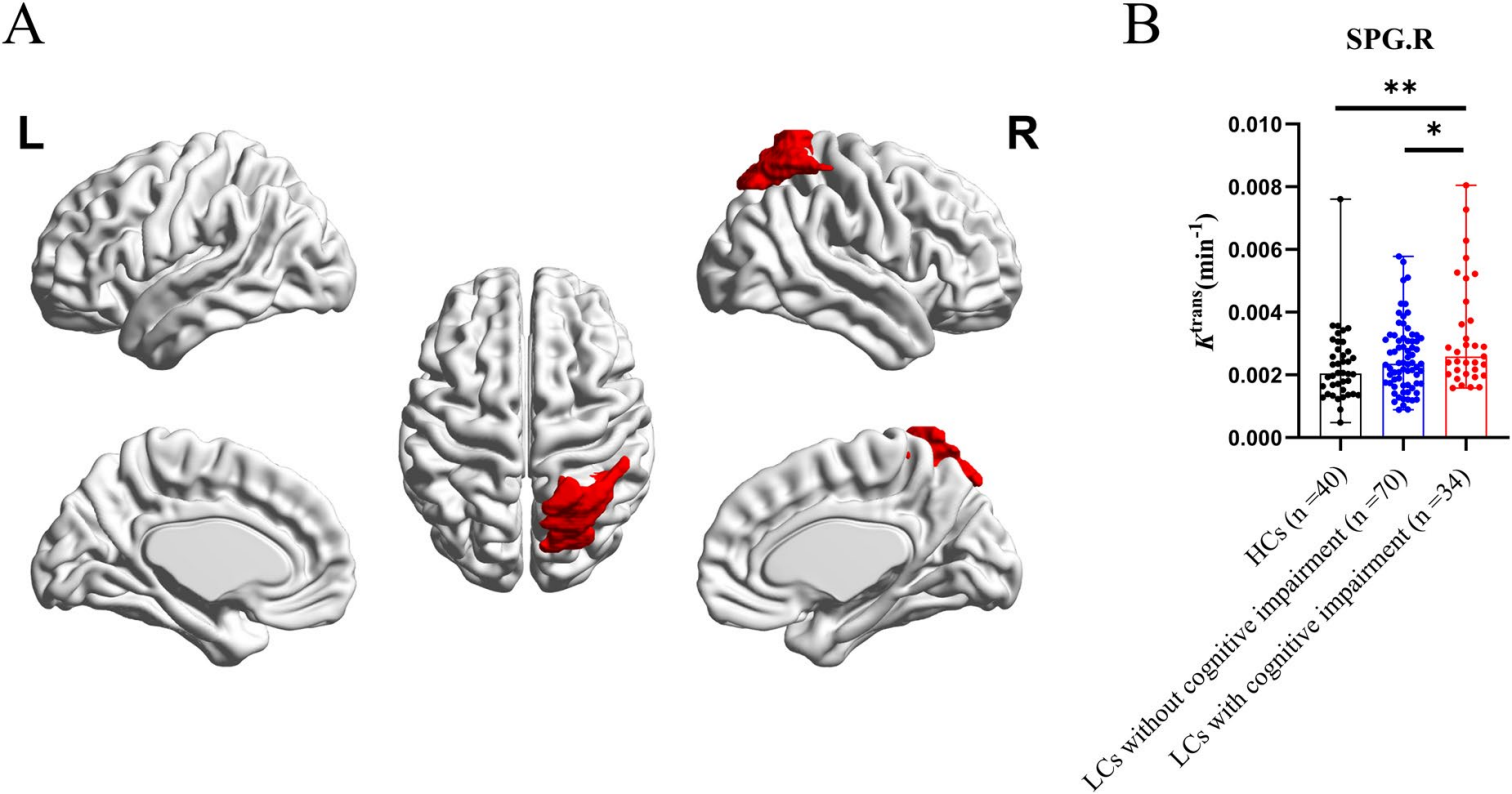
Differences in cortical volume between different groups. (A) The comparison between different groups shows that certain regions, such as the supramarginal and superior frontal cortices, demonstrate varying volumes across the groups, especially between those with cognitive impairment and the healthy control group. (B) Three-dimensional maps of brain regions represent the locations of brain regions with statistical differences (different colors represent different brain regions). Volume(%) represents the cerebral cortex volume normalized to total intracranial capacity (TIV). *p < 0.05, ** *p* < 0.01, *** *p* < 0.001. lh_caudalanteriorcingulate, left caudal anterior-cingulate cortex; lh_superiorfrontal, left superior frontal gyru; lh_supramarginal, left supramarginal gyrus; rh_caudalmiddlefrontal, right caudal middle frontal gyrus; rh_posteriorcingulate, right posterior-cingulate cortex; rh_insula, right insula.

In this study, we first analyzed cortical volume in lung cancer patients and healthy controls. Results are shown in Supplementary Table 2. And then we statistically analyzed the cortical volumes in three groups: lung cancer patients with cognitive impairment (*n* = 34), lung cancer patients without cognitive impairment (*n* = 70), and healthy controls (*n* = 40). The analysis revealed significant differences in cortical volumes across 10 brain regions among the groups (*p* < 0.05). Specifically, patients with cognitive impairment exhibited substantial volume reductions in the left anterior cingulate gyrus, superior frontal gyrus, and supramarginal gyrus, as well as the right middle frontal gyrus, posterior cingulate gyrus, and insula, compared to those without cognitive impairment and the healthy controls (*p* < 0.05) (Figure 3, Supplementary Tables 2 and 3).

**Table 3.** *K*^trans^ difference brain regions in lung cancer patients with cognitive impairment.

Mean *K^trans^* value	LC with cognitive Impairment (*n* = 34)	LC without cognitive Impairment (*n* = 70)	HCs (*n* = 40)	*H*	*P*
SPG.R(×10^-3^)	2.59(2.03,3.73)	2.35(1.72,3.14)	2.04(1.14,2.76)	7.552	0.023

LC, lung cancer；HCs, healthy controls；*H*, by Kruskal–Wallis test.

### 3.4. *K*^trans^ differences between lung cancer patients with and without cognitive impairment and HCs

**Figure 4.**
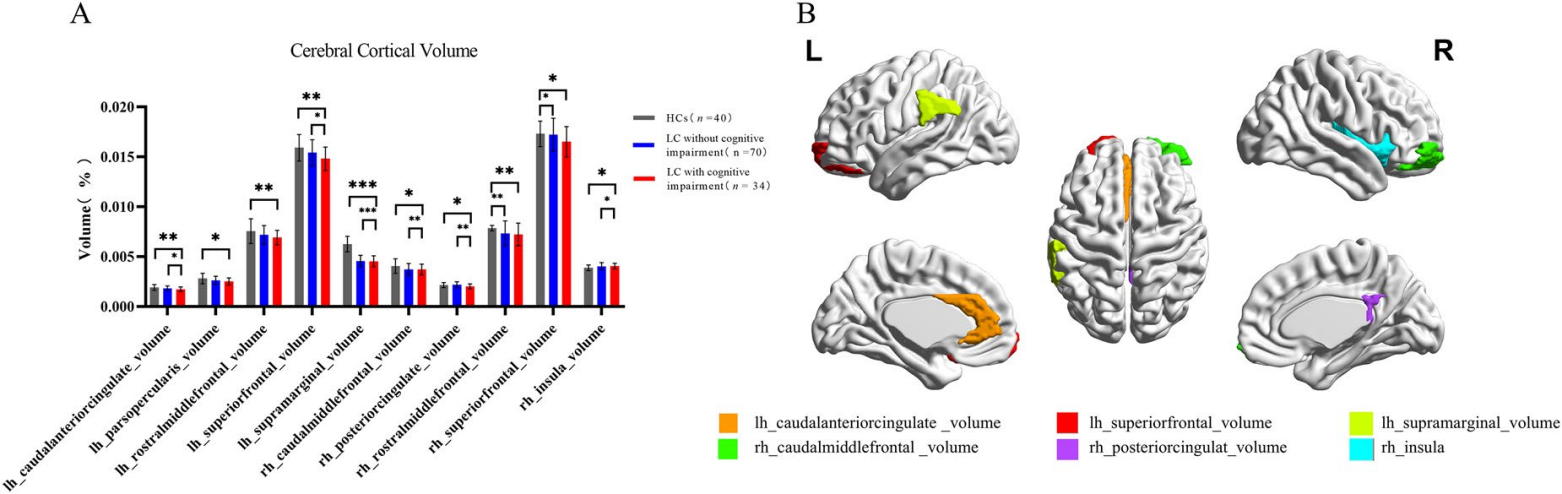
Brain region and three-dimensional maps of the differences in *K*^trans^ between different groups. (A) 3D reconstruction highlighting the right superior parietal gyrus (SPG), where *K^trans^* values were significantly elevated in cognitively impaired patients (red: high permeability). (B) *K*^trans^ values in the right superior parietal gyrus of LC patients with cognitive impairment were significantly higher than those of LC patients without cognitive impairment (Bonferroni-corrected *p* < 0.05). SPG.R, right Superior parietal gyrus, Bonferroni correction was applied across 116 AAL regions. **p < 0.01, ***p < 0.001.

We first analyzed BBB values of lung cancer patients and healthy controls, and the results are shown in Supplementary Table 4. And then we compared 34 lung cancer (LC) patients with cognitive impairment, 70 LC patients without cognitive impairment, and 40 healthy controls. The analysis indicated that the *K*^trans^ values in the right superior parietal gyrus were significantly elevated in the group with cognitive impairment compared to the group without it (Bonferroni-corrected *p* < 0.05). No significant difference was observed between the *K*^trans^ values of the non-cognitive impairment group and the healthy controls (Table 3, Supplementary Table 5, Figure 4, Supplementary Figures 1 and 2).

## 4. Discussion

This study provides the first noninvasive assessment of blood–brain barrier (BBB) permeability in untreated lung cancer patients without brain metastases, linking it to structural brain alterations and cognitive dysfunction. While our cohort included patients across all TNM stages (I–IV), the majority were diagnosed with non-small cell lung cancer (NSCLC), predominantly adenocarcinoma. Notably, small cell lung cancer (SCLC), known for its neurotropic potential and propensity to disrupt the BBB, was underrepresented in our sample. Therefore, our findings are likely more applicable to NSCLC populations, and further research is needed to elucidate histology-specific mechanisms. Using dynamic contrast-enhanced MRI (DCE-MRI), we observed significantly elevated BBB permeability in patients compared to healthy controls, particularly in the right superior parietal gyrus (SPG) of cognitively impaired individuals, where increased *K*^trans^ values correlated with cortical atrophy in regions critical for cognition—including the left anterior cingulate cortex, superior frontal gyrus, and right insula. These structural changes, distinct from prior reports of white matter reductions in small cell lung cancer [11], highlight gray matter vulnerability potentially tied to lung cancer subtypes. Notably, SPG dysfunction, a hub for visuospatial integration and working memory [26–30], may drive cognitive deficits through BBB leakage, aligning with neuroinflammatory pathways observed in Alzheimer’s disease [31,32] and post-chemotherapy brain changes [33–36].

We propose that lung cancer progression disrupts BBB integrity *via* cytokine-mediated inflammation (e.g. TNF-α/IL-6-induced claudin-5 downregulation) [12,13,16,17] and mesenchymal circulating tumor cell (CTC) infiltration, creating a vicious cycle: BBB compromise permits neurotoxic agents to exacerbate cortical atrophy, while microcirculatory dysfunction facilitates CTC adhesion and micrometastasis, further degrading BBB integrity. This bidirectional mechanism may amplify neuropathological burden, bridging cancer-associated inflammation to neurodegenerative processes.

Despite these insights, our study has several limitations: First, a modest single-center sample size, precluded detailed subgroup analyses by specific TNM stages (e.g. IIIA vs. IV) or histological subtypes (e.g. adenocarcinoma vs. squamous cell carcinoma), limiting the generalizability of our findings across all lung cancer categories. Second, although healthy controls were carefully matched for age, sex, and smoking history, and all cortical volumes were normalized to TIV with age included as a covariate in analyses, residual confounding due to undetected age-related cerebral atrophy cannot be entirely ruled out. Third, reliance on the MoCA scale, while validated in oncology populations, may lack sensitivity to subtle cognitive deficits. Fourth, the cross-sectional design precludes causal inference regarding the directionality between BBB disruption and cognitive impairment. Finally, the potential impact of immunotherapy on BBB integrity and cognitive function warrants attention. As immune checkpoint inhibitors (e.g. anti-PD-1/PD-L1 agents) are increasingly used in advanced lung cancer, their effects on neuroinflammation and BBB permeability remain poorly understood. Future studies should investigate whether immunotherapy, alone or in combination with chemotherapy, exacerbates or ameliorates BBB disruption and cognitive decline. Such insights could guide tailored therapeutic strategies that preserve cognitive function while achieving tumor control.

Notwithstanding these limitations, future multi-center studies with larger cohorts, comprehensive neuropsychological batteries (e.g. Trail Making Test), explicit histology-stratified sampling, and experimental validation (e.g. *in vitro* BBB models exposed to CTCs/cytokines) are needed to refine K^trans^ as a prognostic biomarker for cognitive decline. Clinically, integrating DCE-MRI into routine assessments could identify high-risk patients for early neuroprotective therapies (e.g. anti-IL-6 agents) or cognitive rehabilitation. Moreover, BBB permeability evaluation may extend to other cancers (e.g. breast, colorectal [8,34], offering a universal biomarker for neurotoxicity monitoring. By addressing these gaps, our findings lay groundwork for personalized interventions that balance tumor control with cognitive preservation in lung cancer care.

## Supplementary Material

Supplemental Material

Supplemental Material

Supplemental Material

Supplementary_Clean.docx

## Data Availability

The data that support the findings of the current study may be requested from the corresponding author upon reasonable request.

## Abbreviations

AAL: Anatomical Automatic Labeling
AIF: arterial input function
aLC: advanced-stage lung cancer
BBB: blood–brain barrier
CRCI: cancer-related cognitive impairment
CYFRA21-1: Cytokeratin 19 fragment
CEA: carcinoembryonic antigen
CTCs: circulating tumor cells
DCE-MRI: Dynamic enhancement MRI
eLC: early-stage lung cancer
FLAIR: fluid-attenuated inversion recovery
HC: healthy controls
IL-: interleukin
KPS: Karnofsky Performance Scale
LC: lung cancer
MoCA: Montreal Cognitive Assessment
MNI: Montreal Neurological Institute
NSE: neuron-specific enolase
ROI: region of interest
SCC: squamous cell carcinoma antigen
SPG: the right superior parietal gyrus
TNF-α: tumor necrosis factor alpha
TIV: total intracranial capacity.
