# Supplementary material for: Unveiling the cognitive fog in lung cancer patients: non-invasive exploration of blood–brain barrier disruption and brain structural changes
Source: Ann Med. 2026 Jun 18;58(1):2662776. doi: 10.1080/07853890.2026.2662776 (PMC13288549; doi:10.1080/07853890.2026.2662776)
Supplement: Supplemental Material [file IANN_A_2662776_SM9933.docx]

Supplementary figure 1 Comparison of BBB Differences Between Healthy Controls and Lung Cancer Patients with Cognitive Impairment. L, left; R, right; DCG, Median cingulate and paracingulate gyri; PCG, Posterior cingulate gyrus; CAL, Calcarine fissure and surrounding cortex; MOG, Middle occipital gyrus; SOG, Superior occipital gyrus; SMG, Supramarginal gyrus; ANG, Angular gyrus

Supplementary figure 2 Three-dimensional maps of brain regions represent the locations of brain regions with statistical differences (different colors represent different brain regions).


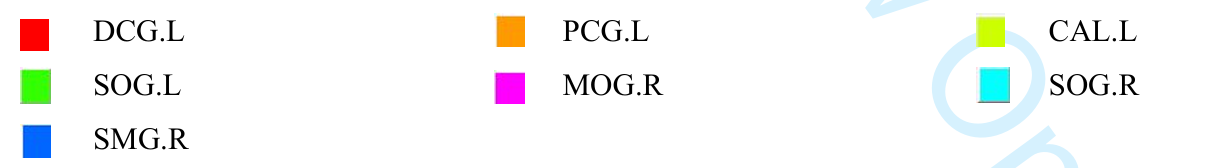


L, left; R, right; DCG, Median cingulate and paracingulate gyri; PCG, Posterior cingulate gyrus; CAL, Calcarine fissure and surrounding cortex; MOG, Middle occipital gyrus; SOG, Superior occipital gyrus; SMG, Supramarginal gyrus; ANG, Angular gyrus
