# Supplementary figures and images for: Unveiling the cognitive fog in lung cancer patients: non-invasive exploration of blood–brain barrier disruption and brain structural changes
Source: Ann Med. 2026 Jun 18;58(1):2662776. doi: 10.1080/07853890.2026.2662776 (PMC13288549; doi:10.1080/07853890.2026.2662776)

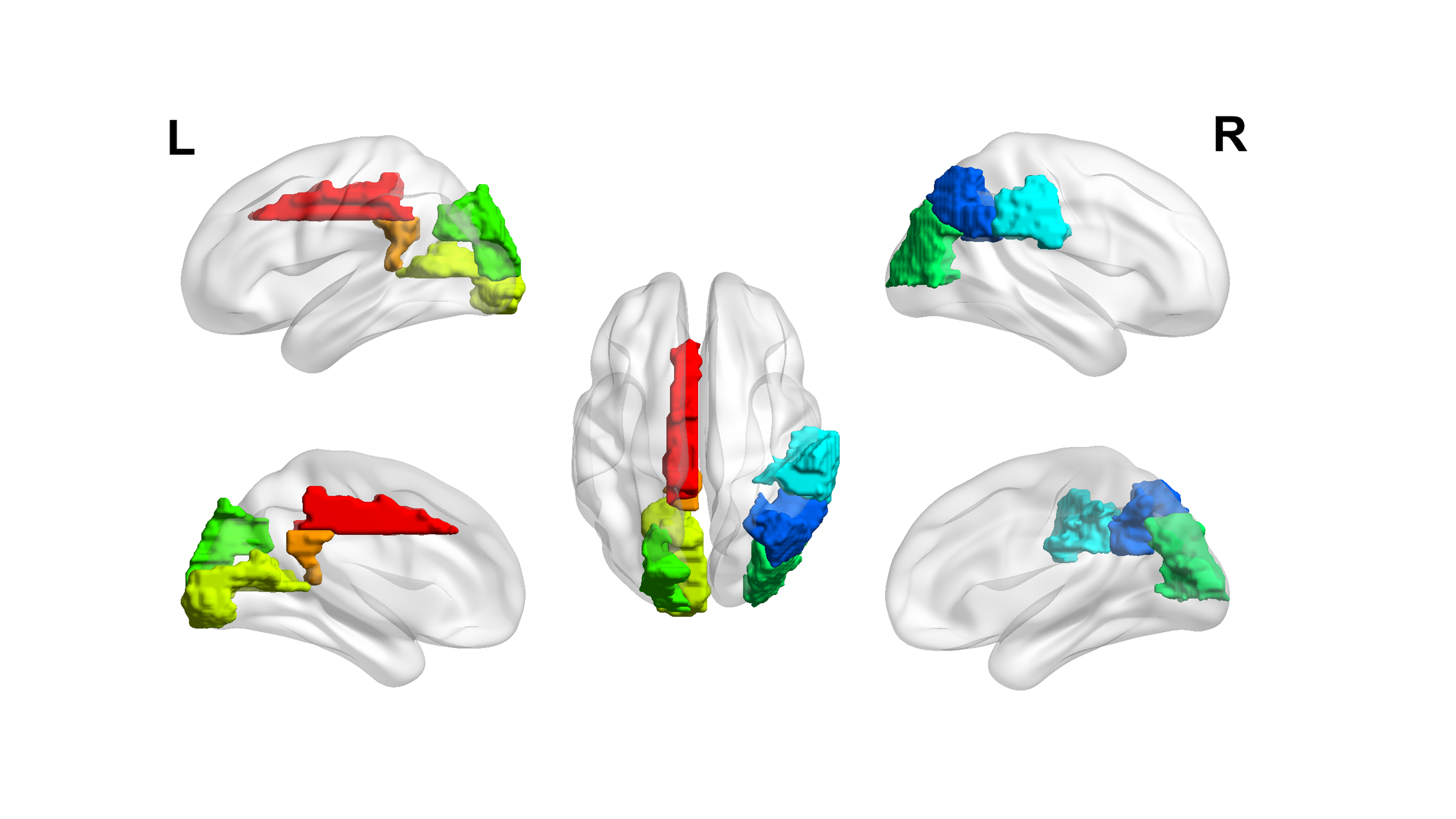

Supplement: Supplemental Material [file IANN_A_2662776_SM9107.tif]

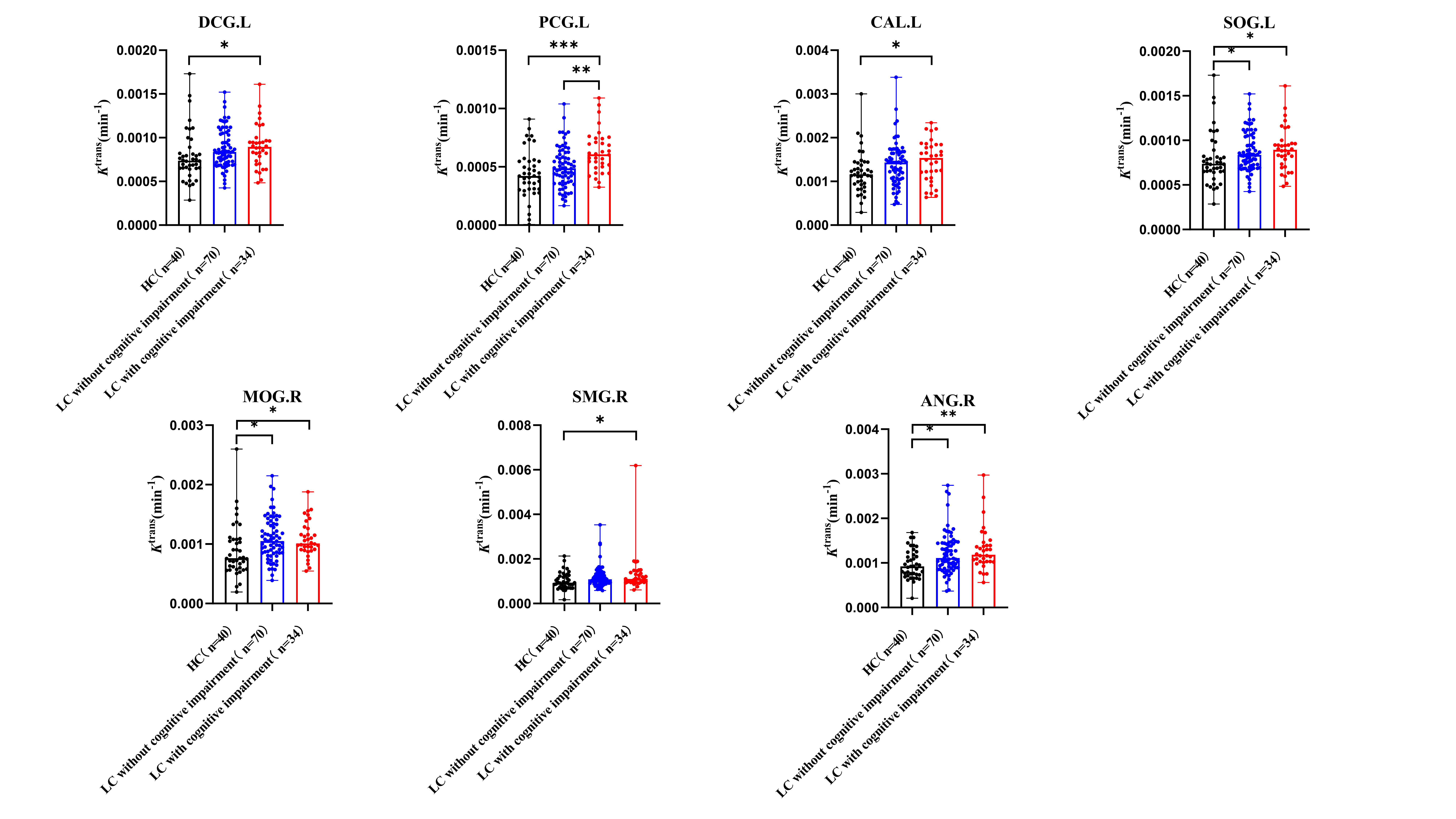

Supplement: Supplemental Material [file IANN_A_2662776_SM9106.tif]
