# Supplementary material for: Unveiling the cognitive fog in lung cancer patients: non-invasive exploration of blood–brain barrier disruption and brain structural changes
Source: Ann Med. 2026 Jun 18;58(1):2662776. doi: 10.1080/07853890.2026.2662776 (PMC13288549; doi:10.1080/07853890.2026.2662776)
Supplement: Supplementary_Clean.docx [file IANN_A_2662776_SM8604.docx]

Supplementary Table 1 Brain routine sequence scanning parameters

| Sequence | T_1_WI | T_2_WI | DWI | Flair | T_1_ Enhancement |
| --- | --- | --- | --- | --- | --- |
| TR (ms) | 162 | 5000 | 4000 | 9000 | 190 |
| TE (ms) | 100 | 120 | 67.2 | 120 | 67.2 |
| FOV (mm) | 240×240 | 240×240 | 240×240 | 240×240 | 240×240 |
| Matrix | 195×210 | 284×284 | 128×128 | 224×224 | 195×210 |
| Thickness(mm) | 6 | 6 | 6 | 6 | 6 |
| Layer spacing (mm) | 1 | 1 | 1 | 1 | 1 |
| Scanning time | 46秒 | 40秒 | 33秒 | 117秒 | 150秒 |

Note: T1WI: T1 weighted imaging; T2WI: T2 weighted imaging; DWI: diffusion weighted imaging; Flair: liquid attenuation inversion recovery imaging

|  | Healthy Controls (n=40) | | Lung Cancer Patients (n=104) | |  |  |
| --- | --- | --- | --- | --- | --- | --- |
| Brain Region | *‾x* | *s* | *‾x* | *s* | *t* | *p* |
| lh_caudalanteriorcingulate_volume | 1.92E-03 | 2.72E-04 | 1.81E-03 | 2.44E-04 | 2.568 | 0.011 |
| lh_entorhinal_volume | 1.17E-03 | 2.59E-04 | 1.10E-03 | 1.90E-04 | 1.989 | 0.049 |
| lh_parsopercularis_volume | 2.85E-03 | 5.33E-04 | 2.62E-03 | 3.92E-04 | 2.801 | 0.006 |
| lh_parstriangularis_volume | 2.91E-03 | 5.59E-04 | 2.71E-03 | 4.46E-04 | 2.288 | 0.024 |
| lh_rostralmiddlefrontal_volume | 2.23E-03 | 2.94E-04 | 2.17E-03 | 2.98E-04 | 2.536 | 0.012 |
| lh_superiorfrontal_volume | 1.59E-02 | 1.37E-03 | 1.53E-02 | 1.32E-03 | 2.653 | 0.009 |
| rh_caudalmiddlefrontal_volume | 4.03E-03 | 7.05E-04 | 3.73E-03 | 5.79E-04 | 2.701 | 0.008 |
| rh_fusiform_volume | 1.08E-03 | 1.99E-04 | 1.03E-03 | 2.12E-04 | 2.102 | 0.037 |

Supplementary table 2 Comparison of Cerebral Cortex Volume between Healthy Controls and Lung Cancer Patients

Comparison of cerebral cortex volume between healthy controls and lung cancer patients. The data is the ratio of the cerebral cortex surface area to the total brain volume, to eliminate the influence of differences in head size between individuals. Values are expressed as x ± s; a two-sample t-test was used to obtain P values, with P<0.05 considered statistically significant.

lh_caudalanteriorcingulate_volume, Left anterior cingulate cortex volume; lh_entorhinal_volume, Left entorhinal cortex; lh_parsopercularis_volume, Left insular cortex volume; lh_parstriangularis_volume, Left inferior frontal gyrus triangular part volume; lh_rostralmiddlefrontal_volume, Left rostral middle frontal cortex volume; lh_superiorfrontal_volume, Left superior frontal gyrus volume; rh_caudalmiddlefrontal_volume, Right middle frontal gyrus volume; rh_fusiform_volume, Fusiform nucleus cortex.

Supplementary table 3 Comparison of Cerebral Cortex Volume among Healthy Controls, Lung Cancer Patients without Cognitive Impairment, and Lung Cancer Patients with Cognitive Impairment

| Brain Region | Healthy Controls (n=40) | | Lung Cancer without Cognitive Impairment Group (n=70) | | Lung Cancer with Cognitive Impairment Group (n=34) | | *F* | *P* |
| --- | --- | --- | --- | --- | --- | --- | --- | --- |
|  | *‾x* | *s* | *‾x* | *s* | *‾x* | *s* |  |  |
| lh_caudalanteriorcingulate_volume | 1.91E-03 | 2.79E-04 | 1.83E-03 | 2.29E-04 | 1.72E-03 | 2.32E-04 | 5.568 | 0.004 |
| lh_parsopercularis_volume | 2.80E-03 | 5.21E-04 | 2.63E-03 | 3.90E-04 | 2.51E-03 | 3.53E-04 | 4.670 | 0.011 |
| lh_rostralmiddlefrontal_volume | 7.55E-03 | 1.24E-03 | 7.17E-03 | 9.39E-04 | 6.90E-03 | 7.26E-04 | 4.130 | 0.018 |
| lh_superiorfrontal_volume | 1.59E-02 | 1.32E-03 | 1.54E-02 | 1.31E-03 | 1.48E-02 | 1.18E-03 | 6.283 | 0.002 |
| lh_supramarginal_volume | 6.25E-03 | 7.74E-04 | 4.55E-03 | 5.73E-04 | 4.52E-03 | 5.56E-04 | 105.3 | 0.000 |
| rh_caudalmiddlefrontal_volume | 4.05E-03 | 7.22E-04 | 3.72E-03 | 5.90E-04 | 3.70E-03 | 5.43E-04 | 4.240 | 0.016 |
| rh_posteriorcingulate_volume | 2.14E-03 | 2.51E-04 | 2.18E-03 | 2.89E-04 | 2.01E-03 | 2.48E-04 | 4.550 | 0.012 |
| rh_rostralmiddlefrontal_volume | 1.85E-03 | 2.80E-04 | 7.33E-03 | 1.24E-03 | 7.22E-03 | 1.14E-03 | 394.4 | 0.000 |
| rh_superiorfrontal_volume | 1.73E-02 | 1.28E-03 | 1.72E-02 | 1.66E-03 | 1.65E-02 | 1.52E-03 | 3.239 | 0.042 |
| rh_insula_volume | 3.87E-03 | 2.84E-04 | 4.03E-03 | 3.59E-04 | 4.05E-03 | 2.69E-04 | 3.451 | 0.034 |

Comparison of cerebral cortex volume among lung cancer patients with cognitive impairment, without cognitive impairment, and healthy controls. The data is expressed as x ± s; a one-way ANOVA was used to obtain P values, with P<0.05 considered statistically significant. Multiple comparisons were made using the LSD method.

lh_caudalanteriorcingulate_volume, Left anterior cingulate cortex volume; lh_parsopercularis_volume, Left insular cortex volume; lh_rostralmiddlefrontal_volume, Left rostral middle frontal cortex volume; lh_superiorfrontal_volume, Left superior frontal gyrus volume; lh_supramarginal_volume, Left supramarginal gyrus volume; rh_caudalmiddlefrontal_volume, Right middle frontal gyrus volume; rh_posteriorcingulate_volume, Right posterior cingulate cortex volume; rh_rostralmiddlefrontal_volume, Right rostral middle frontal cortex volume; rh_superiorfrontal_volume, Right superior frontal gyrus volume; rh_insula_volume, Right insular cortex volume.

Supplementary table 4 Comparison of BBB Differences between Healthy Controls and Lung Cancer Patients

|  | Healthy Controls (n=40) | | | Lung Cancer Group (n=104) | | |
| --- | --- | --- | --- | --- | --- | --- |
|  | 25% | Median | 75% | 25% | Median | 75% |
| PreCG.L | 1.00E-03 | 1.45E-03 | 1.88E-03 | 1.28E-03 | 1.60E-03 | 2.02E-03 |
| MFG.L | 1.18E-03 | 1.62E-03 | 2.21E-03 | 1.56E-03 | 2.00E-03 | 2.54E-03 |
| MFG.R | 9.95E-04 | 1.43E-03 | 2.10E-03 | 1.42E-03 | 1.85E-03 | 2.35E-03 |
| IFGoperc.L | 8.11E-04 | 1.05E-03 | 1.41E-03 | 1.04E-03 | 1.23E-03 | 1.42E-03 |
| IFGoperc.R | 9.32E-04 | 1.08E-03 | 1.69E-03 | 1.17E-03 | 1.43E-03 | 1.67E-03 |
| IFGtriang.L | 9.01E-04 | 1.24E-03 | 1.70E-03 | 1.27E-03 | 1.49E-03 | 1.90E-03 |
| SFGmed.L | 1.69E-03 | 2.27E-03 | 2.95E-03 | 2.15E-03 | 2.55E-03 | 3.08E-03 |
| DCG.L | 6.50E-04 | 7.37E-04 | 8.38E-04 | 7.16E-04 | 8.46E-04 | 1.02E-03 |
| DCG.R | 2.65E-04 | 3.50E-04 | 4.62E-04 | 3.55E-04 | 4.19E-04 | 5.47E-04 |
| PCG.L | 3.09E-04 | 4.21E-04 | 5.47E-04 | 4.22E-04 | 5.28E-04 | 6.61E-04 |
| CAL.L | 9.37E-04 | 1.16E-03 | 1.43E-03 | 1.05E-03 | 1.43E-03 | 1.71E-03 |
| CUN.L | 1.84E-03 | 2.14E-03 | 2.39E-03 | 2.00E-03 | 2.40E-03 | 2.82E-03 |
| LING.L | 3.90E-04 | 5.27E-04 | 6.27E-04 | 4.56E-04 | 6.24E-04 | 7.57E-04 |
| LING.R | 3.27E-04 | 5.40E-04 | 7.02E-04 | 4.55E-04 | 6.16E-04 | 7.62E-04 |
| SOG.L | 7.10E-04 | 1.05E-03 | 1.40E-03 | 9.46E-04 | 1.23E-03 | 1.54E-03 |
| SOG.R | 6.57E-04 | 8.10E-04 | 1.13E-03 | 8.46E-04 | 1.06E-03 | 1.31E-03 |
| MOG.L | 3.88E-04 | 5.28E-04 | 8.13E-04 | 5.79E-04 | 7.04E-04 | 9.14E-04 |
| MOG.R | 5.96E-04 | 7.60E-04 | 1.08E-03 | 8.65E-04 | 1.05E-03 | 1.35E-03 |
| IOG.L | 5.25E-04 | 6.33E-04 | 1.03E-03 | 6.80E-04 | 8.86E-04 | 1.20E-03 |
| IOG.R | 7.20E-04 | 9.89E-04 | 1.55E-03 | 9.40E-04 | 1.38E-03 | 1.88E-03 |
| FFG.R | 6.03E-04 | 7.30E-04 | 9.59E-04 | 6.90E-04 | 8.45E-04 | 1.06E-03 |
| PoCG.L | 1.03E-03 | 1.17E-03 | 1.79E-03 | 1.20E-03 | 1.59E-03 | 2.09E-03 |
| PoCG.R | 1.08E-03 | 1.42E-03 | 1.73E-03 | 1.25E-03 | 1.76E-03 | 2.14E-03 |
| SPG.L | 9.67E-04 | 1.37E-03 | 1.76E-03 | 1.25E-03 | 1.64E-03 | 2.40E-03 |
| SPG.R | 1.49E-03 | 2.04E-03 | 2.76E-03 | 1.87E-03 | 2.42E-03 | 3.26E-03 |
| IPL.L | 5.88E-04 | 8.51E-04 | 1.13E-03 | 7.26E-04 | 1.05E-03 | 1.48E-03 |
| IPL.R | 9.65E-04 | 1.19E-03 | 1.54E-03 | 1.12E-03 | 1.41E-03 | 1.76E-03 |
| SMG.L | 5.51E-04 | 7.18E-04 | 9.36E-04 | 7.03E-04 | 8.76E-04 | 1.09E-03 |
| SMG.R | 7.47E-04 | 9.21E-04 | 1.23E-03 | 9.48E-04 | 1.10E-03 | 1.39E-03 |
| ANG.L | 5.06E-04 | 6.09E-04 | 8.39E-04 | 5.89E-04 | 8.11E-04 | 1.11E-03 |
| ANG.R | 7.39E-04 | 9.23E-04 | 1.20E-03 | 9.31E-04 | 1.19E-03 | 1.46E-03 |
| TPOsup.L | 1.08E-03 | 1.32E-03 | 1.60E-03 | 1.31E-03 | 1.78E-03 | 2.46E-03 |
| TPOsup.R | 1.11E-03 | 1.58E-03 | 1.93E-03 | 1.51E-03 | 1.89E-03 | 2.37E-03 |
| MTG.L | 5.66E-04 | 7.43E-04 | 9.00E-04 | 7.11E-04 | 8.74E-04 | 1.09E-03 |
| MTG.R | 7.18E-04 | 8.88E-04 | 1.13E-03 | 8.94E-04 | 1.04E-03 | 1.25E-03 |
| TPOmid.L | 9.85E-04 | 1.40E-03 | 1.92E-03 | 1.41E-03 | 1.93E-03 | 2.47E-03 |
| TPOmid.R | 1.33E-03 | 1.77E-03 | 2.39E-03 | 1.77E-03 | 2.28E-03 | 2.87E-03 |
| ITG.L | 5.83E-04 | 9.04E-04 | 1.24E-03 | 8.10E-04 | 1.14E-03 | 1.41E-03 |
| ITG.R | 8.00E-04 | 1.04E-03 | 1.59E-03 | 1.13E-03 | 1.33E-03 | 1.77E-03 |
| Cerebelum_Crus1_L | 1.37E-03 | 1.74E-03 | 1.94E-03 | 1.46E-03 | 1.87E-03 | 2.55E-03 |
| ktransGray | 5.40E-04 | 6.48E-04 | 7.72E-04 | 6.18E-04 | 7.19E-04 | 8.27E-04 |

In the comparison between lung cancer patients and healthy controls for BBB, the data is represented as (25%, median, 75%); the P value is obtained using the Mann-Whitney U test; a P value < 0.05 indicates a statistically significant difference.

PreCG, precentral gyrus; MFG, Middle frontal gyrus; IFGoperc: Inferior frontal gyrus, opercular part; IFGtriang, Inferior frontal gyrus, triangular part; SFGmed, Superior frontal gyrus, medial; DCG, Median cingulate and paracingulate gyri; PCG, Posterior cingulate gyrus; CAL, Calcarine fissure and surrounding cortex; CUN, cuneus; LING, Lingual gyrus; SOG, Superior occipital gyrus; MOG, Middle occipital gyrus; IOG, Inferior occipital gyrus; FFG, Fusiform gyrus; PoCG, Postcentral gyrus; SPG, Superior parietal gyrus; IPL, Inferior parietal, but supramarginal and angular gyrus; SMG, Supramarginal gyrus; ANG, Angular gyrus; TPOsup, Temporal pole: superior temporal gyrus; MTG, Middle temporal gyrus; TPOmid, Temporal pole: middle temporal gyrus; ITG, Inferior temporal gyrus; Cerebelum_Crus1_L, left cerebellar crus 1.

Supplementary table 5 Comparison of BBB Differences Between Healthy Controls and Lung Cancer Patients with Cognitive Impairment

|  | Healthy Controls (n=40) | | | Lung Cancer without Cognitive Impairment Group (n=70) | | | Lung Cancer with Cognitive Impairment Group (n=34) | | |
| --- | --- | --- | --- | --- | --- | --- | --- | --- | --- |
|  | 25% | Median | 75% | 25% | Median | 75% | 25% | Median | 75% |
| Cingulum_Mid_L | 6.50E-04 | 7.37E-04 | 8.38E-04 | 7.16E-04 | 8.35E-04 | 1.03E-03 | 7.48E-04 | 8.94E-04 | 9.64E-04 |
| Cingulum_Post_L | 3.09E-04 | 4.21E-04 | 5.47E-04 | 3.70E-04 | 4.87E-04 | 6.00E-04 | 5.04E-04 | 6.07E-04 | 7.34E-04 |
| Calcarine_L | 9.37E-04 | 1.16E-03 | 1.43E-03 | 1.07E-03 | 1.43E-03 | 1.67E-03 | 1.11E-03 | 1.53E-03 | 1.83E-03 |
| Occipital_Sup_R | 6.57E-04 | 8.10E-04 | 1.13E-03 | 8.32E-04 | 1.05E-03 | 1.27E-03 | 8.48E-04 | 1.07E-03 | 1.34E-03 |
| Occipital_Mid_R | 5.96E-04 | 7.60E-04 | 1.08E-03 | 8.52E-04 | 1.05E-03 | 1.34E-03 | 9.08E-04 | 1.01E-03 | 1.24E-03 |
| SupraMarginal_R | 7.47E-04 | 9.21E-04 | 1.23E-03 | 9.20E-04 | 1.08E-03 | 1.31E-03 | 9.55E-04 | 1.10E-03 | 1.38E-03 |
| Angular_R | 7.39E-04 | 9.23E-04 | 1.20E-03 | 8.96E-04 | 1.11E-03 | 1.45E-03 | 1.03E-03 | 1.18E-03 | 1.39E-03 |

The data is presented as (25%, median, 75%), and statistical analysis is performed using the Kruskal-Wallis test; α = 0.05 is set as the significance level for the test, and when P < 0.05, the difference is considered statistically significant. Multiple comparisons are corrected using the Bonferroni correction.

Supplementary figure 1 Comparison of BBB Differences Between Healthy Controls and Lung Cancer Patients with Cognitive Impairment.

L, left; R, right; DCG, Median cingulate and paracingulate gyri; PCG, Posterior cingulate gyrus; CAL, Calcarine fissure and surrounding cortex; MOG, Middle occipital gyrus; SOG, Superior occipital gyrus; SMG, Supramarginal gyrus; ANG, Angular gyrus

Supplementary figure 2 Three-dimensional maps of brain regions represent the locations of brain regions with statistical differences (different colors represent different brain regions).

| 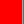 | DCG.L | 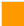 | PCG.L | 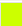 | CAL.L |
| --- | --- | --- | --- | --- | --- |
| 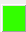 | SOG.L | 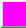 | MOG.R | 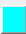 | SOG.R |
| 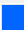 | SMG.R |  |  |  |  |

L, left; R, right; DCG, Median cingulate and paracingulate gyri; PCG, Posterior cingulate gyrus; CAL, Calcarine fissure and surrounding cortex; MOG, Middle occipital gyrus; SOG, Superior occipital gyrus; SMG, Supramarginal gyrus; ANG, Angular gyrus
